# Audience immersion: validating attentional and physiological measures against self-report

**DOI:** 10.1186/s41235-023-00475-0

**Published:** 2023-04-19

**Authors:** Hugo Hammond, Michael Armstrong, Graham A. Thomas, Iain D. Gilchrist

**Affiliations:** 1 School of Psychological Science, University of Bristol, 12a Priory Road, Bristol, BS8 1TU UK; 2 British Broadcasting Corporation (BBC) Research and Development, Saltford, UK

**Keywords:** Immersion, Engagement, Audiences, Attention, Media, Narrative

## Abstract

When an audience member becomes immersed, their attention shifts towards the media and story, and they allocate cognitive resources to represent events and characters. Here, we investigate whether it is possible to measure immersion using continuous behavioural and physiological measures. Using television and film clips, we validated dual-task reaction times, heart rate, and skin conductance against self-reported narrative engagement. We find that reaction times to a secondary task were strongly positively correlated with self-reported immersion: slower reaction times were indicative of greater immersion, particularly emotional engagement. Synchrony in heart rate across participants was associated with self-reported attentional and emotional engagement with the story, although we found no such relationship with skin conductance. These results establish both dual-task reaction times and heart rate as candidate measures for the real-time, continuous, assessment of audience immersion.

**Supplementary Information:**

The online version contains supplementary material available at 10.1186/s41235-023-00475-0.

## Significance statement

The average UK adult spends almost a third of their waking hours (5 hours and 40 minutes per day) watching television, film, or other online video content (Ofcom, 2021). One often-desired property of this media is that it elicits immersion: it can captivate viewers, sustain attention, and lead to total envelopment in the on-screen world. Despite the prevalence of media in our lives, however, psychologists and media creators understand relatively little about immersion. Immersion is conventionally measured through retrospective questionnaires, which may not be sensitive to in-the-moment fluctuations. More recently, attempts have been made to assess immersion using continuous neural, behavioural, and physiological measurements. In this study, we aim to validate three measures (dual-task reaction times, heart rate, and skin conductance) against a widely used immersion questionnaire: the Narrative Engagement Scale. We find that dual-task reaction times and synchrony in heart rate are strongly related to attentional and emotional engagement with the story. These results may allow researchers and creative industry professionals to better understand continuous fluctuations in immersion. This methodology could be applied throughout various stages of the media development process, for example to pre-screen versions of a scene, or even to provide real-time dynamic feedback to broadcasters or performers.

## Introduction

Imagine you are watching your favourite film or television programme: your heart races, your eyes are glued to the screen, and you fail to notice that several hours have passed while you are completely absorbed in the narrative. This is immersion, which can be defined as an individual’s experience of ‘*a state of deep mental involvement in which their cognitive processes (with or without sensory stimulation) cause a shift in their attentional state such that one may experience dissociation from the awareness of the physical world*’ (Agrawal et al., 2020). This definition shares similarities with other concepts, for example the dissociation from real towards virtual worlds experienced in presence (Sanchez-Vives & Slater, 2005) and the deep mental involvement experienced in transportation (Green & Brock, 2000), narrative engagement (Busselle & Bilandzic, 2009), and narrative absorption (Hakemulder et al., 2017). While immersion is a broad term which may apply to a wider variety of media (Agrawal et al., 2020), here we will focus exclusively on immersion within film and television.

Immersion is dynamic and may fluctuate over the course of an experience, changing from deep mental involvement towards mind-wandering and disengagement (Esterman & Rothlein, 2019; Song et al., 2021). Content creators understand that it is infeasible to maintain an ‘*edge of the seat*’ level of immersion indefinitely, and explicitly design media with the intention that immersion rises and falls (Pearlman, 2015). However, despite the dynamic nature of immersion, much of the research in this area relies on singular retrospective estimates from questionnaires: e.g. Immersive Experience Questionnaire (Rigby et al., 2019). Immersion questionnaires typically measure multiple dimensions, including: attention, perception of time, feelings of being spatially located or transported towards the mediated environment, emotional aspects such as the theory of mind, and absorption within the narrative (see Pianzola, 2021 for a review). Questionnaires then are an attractive measure for capturing the multidimensional nature of immersion. The distinct ways in which individuals become immersed are sometimes classified as different types of immersion: e.g. narrative immersion, emotional immersion, and sensory/perceptual immersion (Nilsson et al., 2016; Ryan, 2001). However, as retrospective estimates, questionnaires are not sensitive to the dynamic nature of immersion and are dependent on the memory of the participant. This leaves questionnaire estimates vulnerable to memory biases such as primacy and recency effects (Glanzer & Cunitz, 1966).

More recently, alternative attempts to index immersion have been made using a range of techniques, including neural measures such as fMRI or EEG (Baldassano et al., 2018; Cohen & Parra, 2016; Dmochowski et al., 2012; Hasson et al., 2008); physiological measures such as heart rate or skin conductance (Richardson et al., 2020; Sukalla et al., 2015); behavioural paradigms such as dual-task (Bezdek & Gerrig, 2017; Hinde et al., 2018), continuous rating (Tchernev et al., 2021), or thought-listing paradigms (Magliano et al., 1996; Pjesivac et al., 2021); and other measures including eye tracking (Madsen et al., 2021) or body motion (Theodorou et al., 2019). For a longer discussion of these efforts, see Millman et al. (2022). When using these techniques, one major outcome is that audiences often demonstrate synchronous responses in response to the media, which can be seen in neural (Hasson, 2004), behavioural (Madsen et al., 2021), and physiological data (Madsen & Parra, 2022).

Within narrative media, immersion may be achieved as individuals construct mental models to represent characters, events, and emotions (Mar & Oatley, 2008; Thon, 2008; van Laer et al., 2014; Zacks, 2013). Early definitions of immersion almost exclusively focussed on how increasingly sophisticated display properties evoke immersion (Slater, 2003) and recent work has confirmed that immersion increases where lower-level audio-visual features of the content become more veridical (Hinde et al., 2022).

To a cognitive psychologist, the descriptions of immersion will sound very reminiscent of William James' (1890) much quoted definition of attention as *‘taking possession of the mind… of one out of what seems several simultaneously possible objects or trains of thought’*. Here, in Experiment 1, we explore the relationship between attention and immersion directly. There are a wide range of cognitive paradigms to measure attention (see Pashler, 1998) but the dual-task paradigm (Kahneman, 1973) probably most closely captures the non-spatial withdrawal of attentional resources from one task to focus on another, and so that is the focus of Experiment 1.

Dual-task reaction times are a classic and extremely well-established measure of attention within psychology (Kahneman, 1973). In this paradigm, participants complete a primary task (watching a film) alongside a simple secondary task (e.g. responding to an auditory tone). Reaction time to the secondary task is taken to indicate the available cognitive resources for that task. Given a finite amount of cognitive resources, any reduction in cognitive resources to the secondary task suggests that more resources are being allocated to the primary task (Lang & Basil, 1998; see Potter & Bolls, 2012 for a review). Dual-task reaction times have been applied previously within media research (Bezdek & Gerrig, 2017; Hinde et al., 2018, 2022; Lang, 2000; Troscianko et al., 2012); however, no study to date has directly validated the task as a measure of immersion. Dual-task reaction times have the advantage that they can provide moment-to-moment estimates of immersion and are easy and inexpensive to collect.

In this experiment, we also investigate whether synchrony in reaction times (e.g. correlations across participants arising from similar cognitive processing of the media) may be driven by immersion. We note that audience synchrony has been used both in the context of viewer *co-presence* (i.e. multiple audience members in the same room) and in the context of individual audience members viewing alone. In this paper, we are referring to the latter: synchrony arising from audience member’s cognitive processing of the content.

## Experiment 1

In Experiment 1, we explore if the dual-task reaction times task performance is related to immersion as measured by a standard immersion questionnaire. Our aim is to validate dual-task reaction times against questionnaire-based, self-reported immersion. We selected the Narrative Engagement Scale (Busselle & Bilandzic, 2009) as our self-report measure, as it is widely used, designed for film and television content, and assesses dimensions of immersion that may relate to underlying cognitive and emotional processes (attentional focus, emotional engagement, narrative presence, narrative understanding). While the authors of this scale name this concept narrative engagement, we can consider this the degree to which individuals are immersed in a story (Bilandzic et al., 2019). Our design used 7 short clips which were likely to vary in immersion, so we could look at the correlations between dual-task reaction times and narrative engagement scores. A secondary objective of these experiments was to compare the full questionnaire to a single-item question assessing immersion, to determine if it is possible to reduce questionnaire length.

## Methods

### Participants

Experiment 1 consisted of 170 participants. Participants were recruited from the University of Bristol Psychology student population and were reimbursed with course credit. The sample size was selected arbitrarily but was preregistered at https://osf.io/4fjyc. Participants were eligible for the experiment if they were aged 18 or above, had normal or corrected-to-normal vision, had unimpaired hearing, and had English as a first language (or an equivalent level of fluency). Participants were excluded who: did not watch all clips (*n* = 2), did not meet the eligibility criteria (*n* = 1), or had an error rate on the dual-task paradigm above or equal to chance (*n* = 3); leaving a final sample size of *n* = 164 (*M*_age_ = 19.93, SD ± 3.16, 138 female, 25 male, 1 preferred not to say). Note that excluding participants performing below chance deviates from our preregistered exclusion criteria of < 75% correct responses (see Additional file 1: Fig. S6 for a replication of these results following the preregistered exclusion criteria). Upon reflection, we do not think it is appropriate to exclude participants for answering incorrectly, as a higher error rate may simply be a consequence of higher engagement.

### Stimuli

Participants viewed clips from television and film content available on BBC iPlayer. Clips were between 141 and 184 s long and were selected from a range of genres. As clips spanned a range of genres, we expected they would account for a range of participant preferences, and therefore each clip would vary in narrative engagement within each participant. Experiment 1 used 7 clips (see Table 1 for details). Excerpts were selected which would not require any prior context to understand. Clips were presented at 1280 × 720p resolution: the maximum available on BBC iPlayer for most content, and so representative of a typical home viewing environment (note: only a limited amount of content is available in 3840 × 2160p resolution when streaming from a compatible smart television).

**Table 1:** Stimuli

Clip	Length (s)	Series, episode	Timestamp	Genre	Description
*Experiment 1*					
Blue planet II	174	1, 1	03:10–06:04	Nature	Wildlife documentary series, presented and narrated by David Attenborough, exploring the planet’s oceans
The dumping ground	144	1, 1	00:00–02:24	Children’s	Growing up in a care home brings all sorts of tough challenges, but the kids at Ashdene Ridge know that if they stick together, they can get through anything life throws at them
Killing eve	149	3, 1	00:32–03:03	Drama	When a spy tracks down a stylish assassin, the hunter becomes the hunted. A bloody, funny thriller about two women lethally obsessed with each other
Gardener’s world	173	2021, 17	02:23–05:16	Lifestyle	Gardening show packed with good ideas, tips, advice from experts and timely reminders to get the most out of your garden, whatever its size or type
Mortimer & whitehouse: gone fishing	141	1, 1	03:03–05:24	Comedy	Two friends, beautiful places and a good natter. Bob Mortimer and Paul Whitehouse go on a life-affirming, funny journey, sharing their changed outlooks and trying to land a catch
Your home made perfect	160	1, 1	02:27–05:07	Lifestyle	Transforming problematic pads into heavenly homes. Cutting edge technology and innovative architects reveal the design dream ordinary houses could become
Line Of duty	184	1, 1	00:00–03:04	Drama	Bent coppers and the detectives sent to stop them. AC-12 isn’t here to make friends. Will their investigations land them deadly enemies?
*Experiment 2*					
Crazy Rich Asians	160	–	00:00–02:40	Comedy (film)	New Yorker Rachel Chu accompanies her boyfriend, Nick, to his best friend’s wedding in Singapore and discovers that Nick is one of the country’s wealthiest and most sought-after bachelors
Dolittle	222	–	00:00–03:42	Children’s (film)	A doctor with a special talent for talking to animals is called upon to embark on an important quest
The world’s most extraordinary homes	185	1, 1	00:00–03:05	Lifestyle	Award-winning architect Piers Taylor and actress and property enthusiast Caroline Quentin explore extraordinary homes built in mountain locations around the world
Saving lives at sea	191	6, 10	46:40–49:51	Documentary	At Saunton Sands, Devon, the Appledore crew respond to a 999 call from a man whose wife is being blown out to sea on her new paddleboard
Snooker 2022: UK seniors	160	Final	00:00–02:40	Sport	Coverage of the 2022 UK Seniors Snooker Championship
The outlaws	132	1, 1	00:00–02:12	Comedy	Seven very different strangers begin their community payback sentences, renovating a derelict building in Bristol. But can any of these ‘outlaws’ really reform?
The terror	252	1, 1	25:00–29:12	Drama	Autumn, 1846. Two ships seeking the fabled Northwest Passage around Canada get caught in the Arctic ice
The tourist	179	1, 1	03:02–06:01	Drama	When a man wakes up in the Australian outback with no memory, he must use the few clues he has to discover his identity before his past catches up with him
Universe	154	1, 1	03:40–06:04	Science	Since the first star lit up the universe, they have been engines of creation. Professor Brian Cox reveals how, ultimately, stars brought life and meaning to the universe
The wild gardener	164	1, 1	16:30–19:14	Nature	Wildlife cameraman Colin Stafford-Johnson returns home to Ireland on a personal quest to transform his old childhood garden into a haven for native plants and animals

Content is available to view on BBC iPlayer with a UK TV licence. Genre and descriptions were taken from BBC iPlayer; however, as subsets of each episode were selected the genre and description may not fully reflect the content of each clip

Participants watched the content remotely, on their own laptop or desktop computer. This work was conducted during the COVID-19 pandemic, meaning we could not test in a controlled, in-person setting. However, the value of this work is that we were able to collect data from participants in their naturalistic viewing environment. The experimental window was displayed in Fullscreen, and participants were instructed to ensure they were in a quiet location where they would not be disturbed. Participants could listen using either their device’s speakers or headphones.

### Measures

#### Reaction times

Participants heard random high (1000 Hz) and low (600 Hz) tones at 15-s intervals and were required to make a button press response (left shift for a low tone, right shift for a high tone) as soon as they heard the tone. Tones were 1 s length sine waves, and so distinctive from the audio characteristics of the content. Tones were presented at approximately 10% louder (measured using root mean square energy) than the average volume of all clips. Participants were instructed to set their volume to a comfortable listening level and completed 10 practice trials before the main task to ensure they could discern the tones. As tones themselves may necessarily disrupt the experience of immersion, we elected to compromise varying the interval between tones (and reducing predictability), by creating maximal distance between each tone to avoid potential interference.

#### Narrative engagement

Participants completed the Narrative Engagement Scale (Busselle & Bilandzic, 2009) after watching each clip. This 12-item questionnaire is used to assess four dimensions of engagement: attentional focus, emotional engagement, narrative understanding, and narrative presence. Items are rated on a 7-point Likert scale anchored between ‘strongly disagree’ and ‘strongly agree’. We included a single additional question on immersion (‘During the program, I was very immersed’), rated using the same 7-point Likert scale, to assess the relationship between the full narrative engagement scale and a single dimension.

### Design

We had a within-subjects design, where participants watched each clip in a random order. Because of the total number of clips (7 in Experiment 1), it was not possible to fully counterbalance the design and so the clip order was random. The breakdown of clips in each order is provided in Additional file 1: Fig. S1. Experiment 1 was built using PsychoPy 2021.2.3 (Peirce et al., 2019) and hosted online using https://pavlovia.org, with the information sheet, consent form, and final demographic information being hosted separately using Qualtrics (https://www.qualtrics.com/uk/), version January 2022. All data were analysed using R 4.1.1 (R Core Team, 2021).

## Results

### Immersive narratives consume more attentional resources

Mean dual-task reaction times were *M* = 999 ms, SD = 427 ms with *M* = 92.7%, SD = 26.9% correct responses (see Additional file 1: Fig. S2 for an overall distribution of correct and incorrect responses). In subsequent analyses, responses over 3000 ms and incorrect responses were excluded, as in Hinde et al. (2018). Reaction time data were aggregated by participant and clip, to match the granularity of the self-report measures. While reaction times were not normally distributed (Additional file 1:Fig. S2), we did not transform the data as analyses were conducted on means, which following the central limit theorem will conform to a normal distribution. Mean narrative engagement scores were *M* = 4.27, SD = 0.94. For each dimension, this is: attentional focus (*M* = 4.37, SD = 1.75), emotional engagement (*M* = 3.58, SD = 1.61), narrative presence (*M* = 3.90, SD = 0.73), and narrative understanding (*M* = 5.21, SD = 1.44). In subsequent references to ‘narrative engagement’, we describe the mean of all 12 items. When referring to a dimension of narrative engagement, we describe the mean of the subscale items which assess that dimension. The mean single-item immersion rating was *M* = 4.41, SD = 1.83.

**Fig. 1:**
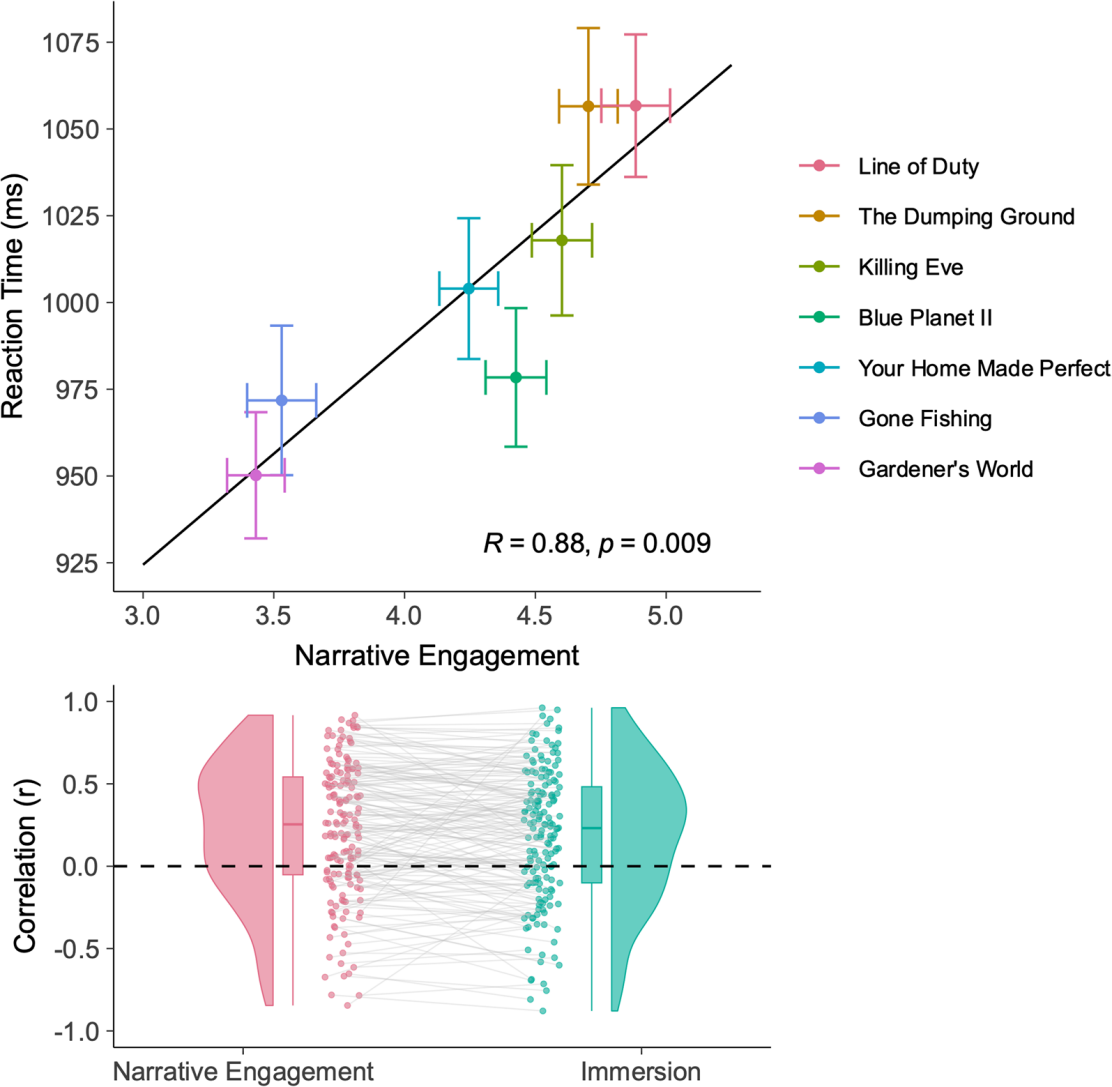
Correlation between narrative engagement and reaction times. Top: Overall correlation between narrative engagement and reaction time for each clip. Error bars denote 95% confidence intervals within each clip. Narrative engagement has been divided by the 12 items in the scale, to create a score between 1 and 7 (note: the factor loading for each item of the scale is unidirectional; Busselle & Bilandzic, 2009), as such it is appropriate to construct a summary score in this way). An overview of the clips used in this experiment can be seen in Table 1. Bottom: Raincloud plot of each participant’s correlation between reaction time and narrative engagement (left) or immersion (right). Each point represents one participant. Boxplots denote median and quartiles, violin plots provide density estimates

Figure 1 shows the overall correlation between reaction time and narrative engagement. To assess this relationship, we fit a linear mixed model to our reaction time data using the ‘lme4’ package in R (Bates et al., 2007). We included a fixed effect of narrative engagement, and participant as a random intercept.^1^ We find that narrative engagement increases reaction time (*b* = 29.24, 95% CI [18.19, 40.26]). Our single-item question of immersion (hereafter referred to as ‘immersion’) also positively influences reaction time (*b* = 14.21, 95% CI [8.71, 19.71]). Individual *p* values are not provided for linear mixed model estimates, due to the problems associated with interpreting *p* values from linear mixed models (Baayen et al., 2008). However, where confidence intervals do not intersect with zero, this can be considered analogous to a significant difference at *p* < 0.05.

To assess whether this relationship was robust within participants, we computed the correlation between reaction time and narrative engagement for each participant and compared this distribution against zero. This approach accounts for individual differences in preference, as the test makes no assumptions about which content participants may rate as most engaging. Using a one-sample, two-tailed t test, we found that this overall distribution was significantly greater than zero: mean *r* = 0.218, *t*(163) = 6.82, *p* = 1.69 × 10^–10^. Similarly, participants’ individual correlation between reaction times and self-reported immersion was significantly greater than zero: mean *r* = 0.185, *t*(163) = 5.85, *p* = 2.58 × 10^–8^.

One interpretation of these data is that the more engaging clips may simply be louder, and as such it may be more difficult to discern reaction time probes, leading to slower responses or increased errors from participants. To address this possibility, we calculated the root mean square energy (RMSE) for each clip’s audio track, as a measure of loudness. Given we only have 7 RMSE values, we are constrained to make comparisons based on those values and have averaged reaction times and tone discrimination error rate per clip for this analysis. RMSE was not significantly correlated with reaction times (*r*(5) = 0.580, *p* = 0.172), but was significantly associated with error rate (*r*(5) = 0.823, *p* = 0.023). As such, we have evidence to conclude that louder clips may be masking the detection of the tones. We therefore included RMSE as a fixed effect in subsequent linear mixed-effects models, to account for this effect of clip volume.

To assess which dimensions of the Narrative Engagement Scale were influencing reaction time, we fitted a linear mixed model to our data using the ‘lme4’ package in R (Bates et al., 2007). We included fixed effects for each dimension of narrative engagement (*attentional focus*, *emotional engagement*, *narrative presence*, *narrative understanding*). We also included fixed effects for *single-item immersion*, *clip order* (which clip was viewed 1st, 2nd 3rd, etc., given the tendency for reaction times to increase over time; Hinde et al., 2018), *clip volume* (RMSE), and for *familiarity* (whether participants had seen the clip before). *Participant* was set as a random slope to account for participant-level differences in average reaction time.

**Fig. 2:**
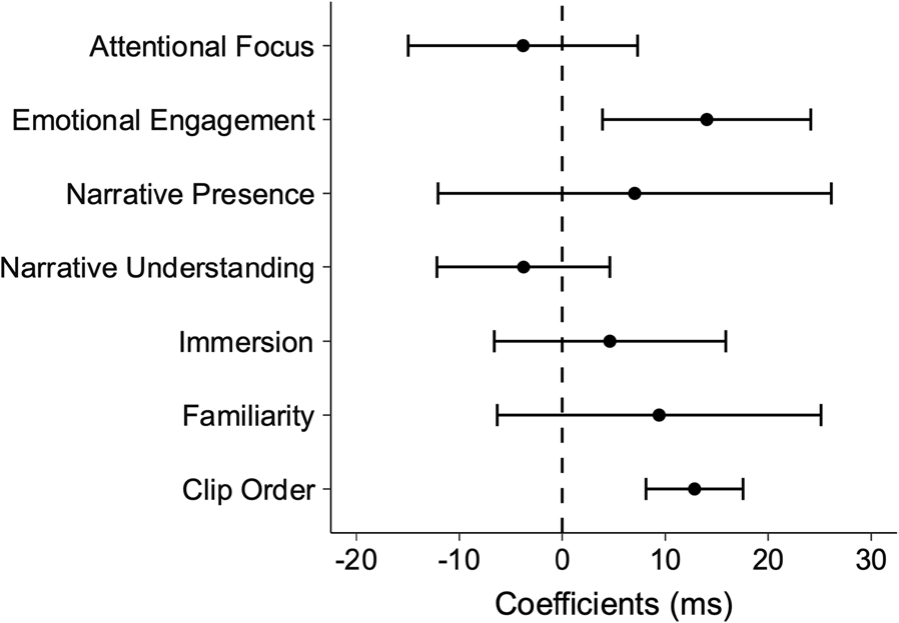
Linear mixed model parameter estimates for reaction time. Error bars denote 95% confidence intervals. Where confidence intervals do not intersect with zero (dashed line), this can be considered analogous to a significant difference at *p* < 0.05. Note that clip volume is not included in this plot as the confidence intervals are exceptionally wide, and make the graph unreadable

As shown in Fig. 2, emotional engagement led to a significant increase in reaction time: *b* = 14.02, 95% CI [3.91, 24.14]. Clip order also significantly increased reaction time: *b* = 12.83, 95% CI [8.13, 17.54]. Narrative presence (*b* = 7.03, 95% CI [− 12.07, 26.13]), narrative understanding (*b* = − 3.78, 95% CI [− 12.18, 4.62]), familiarity (*b* = 9.40, 95% CI [− 6.33, 25.13]), and clip volume (*b* = 314.70, 95% CI [− 980.44, 1610.35]) did not significantly affect reaction time. Interestingly, despite the dual-task paradigm being a measure of attention, attentional focus also did not affect reaction times (*b* = − 3.82, 95% CI[− 14.96, 7.31]). We can conclude therefore that increases in reaction time are predominantly driven by emotional engagement in the story.

We then looked to assess whether synchrony in reaction time was related to narrative engagement. We rely on the most widely used method to measure synchrony: inter-subject correlation (Nastase et al., 2019). For each clip, a correlation matrix between all pairs of participants is produced. We then take an average of each row of that matrix (each participant), which provides a score for each participant of how synchronous they are with all other participants. We find that inter-subject correlation in reaction times (ISC_RT_) was not significantly related to narrative engagement (*r*(5) = 0.554, *p* = 0.197) or immersion (*r*(5) = 0.490, *p* = 0.264). As with mean reaction times, we then looked to assess whether this correlation was robust across participants, by calculating the relationship between individual participant’s ISC_RT_ and narrative engagement. Individual participant’s ISC_RT_ was significantly related to narrative engagement (mean *r* = 0.216, *t*(162) = 6.73, *p* = 2.84 × 10^–10^) and immersion (mean *r* = 0.182, *t*(162) = 5.75, *p* = 4.26 × 10^–08^).

### Familiarity

Additional file S1: Figure S4 (left) presents a breakdown of participant’s familiarity scores in Experiment 1. To assess if familiarity affected our measures, we used Welch’s two-sample, two-tailed *t* tests to account for the unequal sample size and variance between familiar and unfamiliar groups. Participants who had seen any of the series before were more engaged (*M* *unfamiliar* = 4.04, *M* *familiar* = 4.63, *t*(1006) = − 11.06, *p* < 0.001) but did not show differences in reaction times (*M* *unfamiliar* = 985, *M* *familiar* = 989, *t*(939) =  − 0.22, *p* = 0.82). Similarly, participants who had seen the specific clip before were more engaged (*M* *unfamiliar* = 4.14, *M* *familiar* = 4.9, *t*(264) =  − 11.62, *p* < 0.001) but did not show differences in reaction times (*M* *unfamiliar* = 982, *M* *familiar* = 1019, *t*(226) = − 1.45, *p* = 0.15). To summarise, familiarity was associated with higher narrative engagement, but did not affect reaction time.

### Single-item question of immersion indexes the full narrative engagement scale

Finally, we looked to assess whether our single-item question of immersion was related to overall narrative engagement. We found a significant correlation between single-item immersion and the Narrative Engagement Scale: *r*(1146) = 0.797, *p* = 2.2 × 10^–16^. To assess which dimensions immersion was related to, we fit a linear regression predicting immersion from each dimension of narrative engagement. Immersion was predicted by attentional focus (*b* = 0.61, *p* = 2 × 10^–16^), emotional engagement (*b* = 0.28, *p* = 2 × 10^–16^), and narrative presence (*b* = 0.50, *p* = 2 × 10^–16^), but not narrative understanding (*b* = 0.01, *p* = 0.77). This offers a promising indication that most dimensions of engagement (excluding understanding) could be indexed by a single-item questionnaire.

## Discussion

Experiment 1 provides strong evidence that greater levels of immersion are associated with slower dual-task reaction times. This provides strong support for the view that immersion arises from changes in attention (Murray, 1998; Thon, 2008). This is consistent with numerous other findings within the media literature: for example, viewers show attentional synchrony in gaze behaviour (Smith & Henderson, 2008) and viewers are resistant to oculomotor capture by salient visual distractors (Hinde et al., 2017).

A simple way to interpret these results is in terms of an enveloping of perceptual apparatus (Green & Brock, 2000). From this perspective, when immersed, fewer resources are available for the secondary task because they are dedicated towards attending and perceiving the on-screen events. For example, richer visual experiences (such as high dynamic range) lead to slower reaction times on the dual-task paradigm (Hinde et al., 2022). There is some further evidence that larger screens are also more engaging (Troscianko et al., 2012), and that viewing on a television rather than a smartphone is more immersive (Szita & Rooney, 2021). It is possible then that immersion is in part driven by simple visual features such as contrast, luminance, or chrominance.

However, visual properties of the content alone are unlikely to be sufficient to fully explain why more attention is allocated towards engaging stimuli. For example, Bezdek and Gerrig (2017) find that participants are slower to respond to dual-task reaction time probes during moments of higher narrative suspense and provide evidence that simple visual features alone are an inadequate explanation for this. Instead, we may consider immersion as a form of mental simulation, where viewers are occupied with constructing models to represent characters, events, and scenes (Zwaan, 1999). Slower reaction times then may be a consequence of the greater cognitive elaboration arising from processing the narrative, and this is consistent with our result that greater emotional engagement is associated with slower reaction times.

In Experiment 1, familiarity was associated with higher narrative engagement, but did not influence reaction times. This finding intuitively suggests that participants consume more of the content which they find engaging. However, regardless of whether participants have seen the clips before (and therefore may know what to expect), their allocation of attentional resources towards the content remains unchanged. We do note that this experiment did not ask participants how long ago they previously saw the content; participants who recently viewed the content may show differences in attentional orientation.

While dual-task reaction times are able to index the focussed attention arising during immersion, they are not without their own pitfalls. The regular probe intervals may themselves act as a distraction from becoming fully immersed within the media. As an example of this, Hammond et al. (unpublished) find evidence that reaction time probes cause subsequent physiological responses which may be associated with a startle reflex. Further, while providing moment-to-moment estimates, reaction times are not truly continuous (our experiment used an interstimulus interval of 15 s), and as such could not be used to ascertain faster-moving changes in attention and immersion.

## Experiment 2

Experiment 2 looked to further explore the relationship between immersion and two physiological measures: heart rate and skin conductance. These measures have both been widely used within media psychology (Gregersen et al., 2017; Kraj et al., 2020; Richardson et al., 2020; Sukalla et al., 2015); however, they have not yet been validated against self-reported immersion. As physiological measures, they avoid the pitfalls of dual-task reaction times in that they do not disrupt the viewing experience and can easily be sampled at a higher frequency.

Heart rate and skin conductance may also be sensitive to different dimensions of immersion than dual-task reaction times. Heart rate indexes parasympathetic and sympathetic nervous system activity (Levy, 1971), and is known to vary in response to cognitive processing demands (Potter & Bolls, 2012). Skin conductance is one of the few physiological measurements singly innervated by the sympathetic nervous system, is considered a measure of arousal, and is known to vary with the emotional content of a stimulus (Boucsein, 2012). Recent research has found that time-locked correlations in heart rate between participants (synchrony) relate to attention towards narrative content (Madsen & Parra, 2022; Pérez et al., 2021; Stuldreher et al., 2020). Synchrony in skin conductance may additionally relate to the emotional content of the media (Han et al., 2021).

## Methods

### Participants

Experiment 2 recruited 50 participants and used the same eligibility criteria as Experiment 1. Participants were recruited from the University of Bristol student and staff population and were reimbursed with course credit, or £10 financial reimbursement if they were not Psychology students. Two participants were excluded as the equipment failed to capture synchronisation information between the content and physiological responses, leaving a final sample of *n* = 48 (*M*_age_ = 20.22, SD ± 3.16, 40 female, 8 male, 42 right-handed). The sample size was preregistered at https://osf.io/ckx8q.

### Stimuli

Experiment 2 used 10 short clips between 132 and 252 s (see Table 1). Participants viewed the content on a Sony Bravia KD-65ZD9, 142.9 × 80.35 cm screen sitting at a distance of twice the screen height (2H, or 160 cm), as in Hinde et al. (2022).

### Measures

#### Heart rate

Physiological measures were recorded using Biopac MP160 with a sampling rate of 2000 Hz. ECG was recorded using the ECG100C module. Electrodes were placed in lead-III configuration on each collarbone and the lower left rib. Post-processing was done in AcqKnowledge 5.0 to compute heart rate from the interval between each R-R wave and is presented in beats per minute (BPM). Artefacts were identified as regions with no clear R waves and were corrected by linear interpolation from the preceding and following signals.

#### Skin conductance

Skin conductance was recorded using the EDA100C module, and electrodes were placed on the distal phalanges of the index and middle fingers, on the participant’s non-dominant hand. Skin conductance was downsampled to 125 Hz and filtered using a 1 Hz low-pass filter.

#### Narrative engagement

The narrative engagement was obtained through the same procedures as Experiment 1.

### Design

As in Experiment 1, we had a within-subjects design (see Additional file 1: Figure S1 for clip presentation order). Experiment 2 was again built using PsychoPy but conducted in-person in a model living room set-up, complete with television, sofa, and furniture.

## Results

### Immersive narratives synchronise heart rate

For each participant, physiological measures were first averaged over 1-s intervals and difference scores were calculated by subtracting the grand mean for each participant from their scores in each clip. This standardises responses across participants. Mean heart rate was *M* = 75.17, SD = 11.43. Mean narrative engagement scores were (*M* = 4.63, SD = 1.01), and separated into constituent dimensions this was: attentional focus (*M* = 4.80, SD = 1.74), emotional engagement (*M* = 4.17, SD = 1.75), narrative presence (*M* = 4.17, SD = 0.73), narrative understanding (*M* = 5.39, SD = 1.61). For single-item immersion, mean scores were *M* = 4.92, SD = 1.64.

First, we investigated the relationship between heart rate and narrative engagement. Using linear mixed-effects models again, we found a negative relationship between heart rate and narrative engagement (*b* = − 0.057, 95% CI [− 0.100, − 0.014]), and heart rate and immersion (*b* = − 0.027, 95% CI [− 0.053, − 0.002]). Individual participant’s heart rate and engagement correlations were significantly lower than zero for engagement (mean *r* = − 0.123, *t*(46) =  − 2.61, *p* = 0.012) but not immersion (mean *r* = − 0.110, *t*(46) = -1.95, *p* = 0.057), offering some indication that higher self-reported engagement is associated with lower heart rate. For a discussion of heart rate variability, see Additional file 1: Materials S7.

**Fig. 3:**
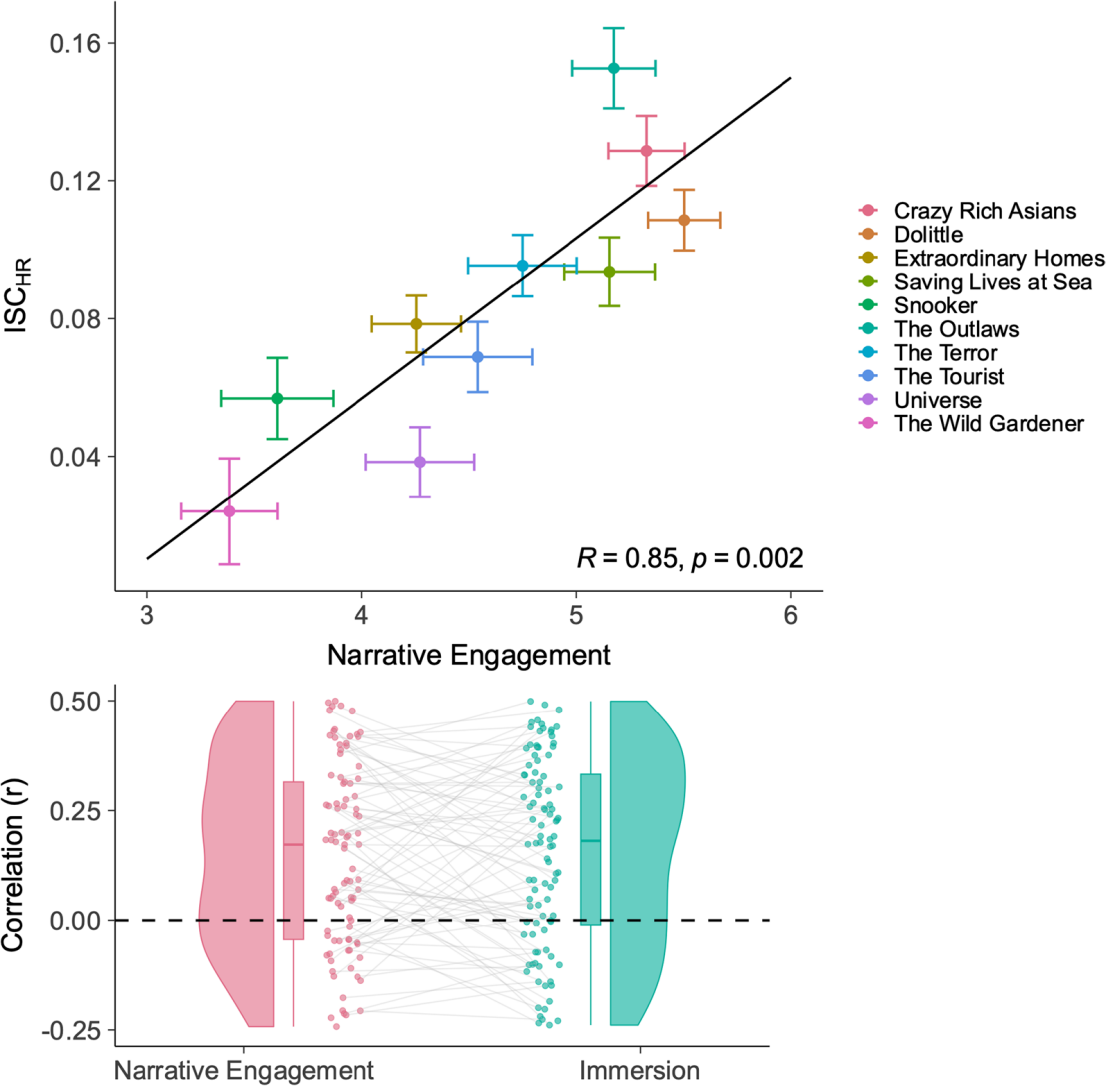
Inter-subject correlations in heart rate. ISC_HR_ is calculated as the correlations in heart rate between each pair of participants. An average is taken for each participant to determine how synchronous they are with other participants. Top: Relationship between narrative engagement and ISC_HR_ for each condition. Error bars denote 95% confidence intervals within each clip. Bottom: Individual correlations between ISC_HR,_ narrative engagement, and immersion. Each point represents one participant. Boxplots denote median and quartiles, violin plots provide density estimates

We find strong evidence that synchronicity in heart rate is a predictor of engagement. Figure 3 (top) plots the relationship between heart rate inter-subject correlation (ISC_HR_) and narrative engagement: narrative engagement increases heart rate synchrony (*b* = 0.026, 95% CI [0.022, 0.029]). A similar relationship was found between ISC_HR_ and immersion: *b* = 0.012, 95% CI [0.010, 0.015]. ISC_HR_ is defined in the same way as ISC_RT_ in Experiment 1.

This relationship between heart rate synchrony and engagement was also robust across participants. Again, we computed correlations between ISC_HR_ and engagement for each participant (Fig. 3: Bottom). Individual participants show significant nonzero relationships between ISC_HR_ and narrative engagement (mean *r* = 0.129, *t*(45) = 7.38, *p* = 2.79 × 10^–9^) as well as between ISC_HR_ and immersion (mean *r* = 0.110, *t*(45) = 6.54, *p* = 4.95 × 10^–8^).

**Fig. 4:**
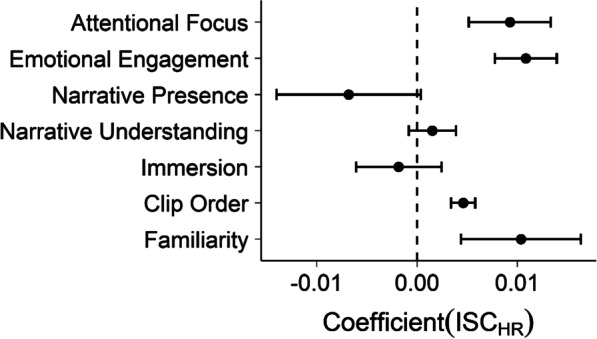
Linear mixed model parameter estimates predicting heart rate inter-subject correlation. Error bars denote 95% confidence intervals. Where confidence intervals do not intersect with zero (dashed line), this can be considered analogous to a significant difference at *p* < .05

As with reaction times above, we then built a linear mixed model, including fixed effects for each dimension of narrative engagement, single-item immersion, clip order, familiarity, and a random intercept for participant. Figure 4 plots these regression estimates.

As shown, attentional focus (*b* = 0.009, 95% CI [0.005, 0.013]), emotional engagement (*b* = 0.010, 95% CI [0.008, 0.014]), clip order (*b* = 0.005, 95% CI [0.003, 0.006]), and familiarity (*b* = 0.010, 95% CI [0.004, 0.012]) significantly increase HR_ISC_, while narrative presence (*b* = -0.007, 95% CI [− 0.014, 0.000]), narrative understanding (*b* = 0.001, 95% CI [− 0.000, 0.004]) and immersion (*b* = − 0.002, 95% CI [− 0.006, 0.002]) do not significantly affect ISC_HR_. Higher narrative engagement drives synchrony between participants' heart rate then, and this is predominantly related to attentional and emotional engagement with the narrative.

### Skin conductance is not related to immersion

Mean skin conductance level for Experiment 2 was *M* = 10.60, SD = 6.08. We did not find a significant relationship between skin conductance level and narrative engagement (*b* = 0.002, 95% CI [− 0.180, 0.186]), or immersion (*b* = − 0.019, 95% CI [− 0.133, 0.095]). Individual participant’s correlations between skin conductance and narrative engagement (mean *r* = − 0.029, *t*(46) =  − 0.58, *p* = 0.57) or immersion (mean *r* = 0.005, *t*(46) = 0.11, *p* = 0.912) were also nonsignificant. Additionally, we did not find evidence of a relationship between inter-subject correlation of skin conductance (ISC_SC_) and narrative engagement (*b* = -0.002, 95% CI [− 0.011, 0.006]) or immersion (*b* = − 0.002, 95% CI [− 0.007, 0.003]). This relationship was also not significant at an individual participant level for engagement (mean *r* = -0.010, *t*(46) = − 0.50, *p* = 0.617) or immersion (mean *r* = − 0.010, *t*(46) = − 0.69,* p* = 0.492).

### Familiarity

In Experiment 2, familiarity was related to higher engagement both when participants had seen any of the series before (*M unfamiliar* = 4.52, *M familiar* = 5.12, *t*(101) = -5.49, *p* = 2.99 × 10^–7^) and when they had seen the specific clip before (*M unfamiliar* = 4.52, *M familiar* = 5.25, *t*(74) = -6.02, *p* = 6.23 × 10^–8^). Heart rate was not affected by familiarity with the series before (*M unfamiliar* = 75.23, *M familiar* = 75.58, *t*(86) = -0.27, *p* = 0.791) or the specific clip (*M unfamiliar* = 75.19, *M familiar* = 75.88, *t*(63) = -0.44, *p* = 0.664). Similarly, skin conductance was not affected by participants seeing any of the series before (*M unfamiliar* = 10.57, *M familiar* = 10.46, *t*(88) = 0.15, *p* = 0.880) or the specific clip before (*M unfamiliar* = 10.62, *M familiar* = 10.17, *t*(74) = 0.65, *p* = 0.516). From this, we can conclude that while participants found familiar content more engaging, this did not affect physiological responses. Additional file 1: Figure S4 (right) presents familiarity scores in Experiment 2.

### Single-item immersion

The same pattern of results was present as in Experiment 1. Our single-item question of immersion was significantly correlated with narrative engagement: *r*(478) = 0.755, *p* = 2.2 × 10^–16^. Again, immersion was predicted by attentional focus (*b* = 0.579, *p* = 2 × 10^–16^), emotional engagement (*b* = 0.150, *p* = 5.88 × 10^–6^), narrative presence (*b* = 0.366, *p* = 4.96 × 10^–7^), but not narrative understanding (*b* = 0.014, *p* = 0.608).

## Discussion

In Experiment 2, we find evidence that self-reported narrative engagement is related to synchronisation across audience’s heart rate. These results add to the growing body of literature which suggests media stimuli can evoke synchronised responses across viewers—in this case at a physiological level. This evidence began with research by Hasson (2004) demonstrating increased inter-subject correlations in brain regions associated with on-screen events (e.g. fusiform gyrus when faces were present on-screen). Since then, synchrony has been found in fMRI (Dmochowski et al., 2014), EEG (Cohen & Parra, 2016; Dmochowski et al., 2012), eye movements (Hutson et al., 2017; Loschky et al., 2015), perception of time (Cohen et al., 2017) and more recently physiological responses such as heart rate and skin conductance (Ardizzi et al., 2020; Czepiel et al., 2021; Golland et al., 2015; Han et al., 2021; Palumbo et al., 2017; Pérez et al., 2021; Stuldreher et al., 2020; Tschacher et al., 2021). This effect of the film content driving a matched response across participants has been termed by some as the ‘tyranny of film’ (Loschky et al., 2015). There is evidence that this synchronous behaviour is modulated by factors including co-presence (viewing together with others; Golland et al., 2015), audience preference (Dmochowski et al., 2014), narrative (Nguyen et al., 2019), emotional content (Dmochowski et al., 2012), attention (Ki et al., 2016), and task performance (Madsen et al., 2021). Here we add to this list: heart rate synchrony may be driven by audience immersion.

While we argue that heart rate synchrony in our study is driven by immersion, a critical reader may suggest that the result is due to an unintended confound, such as laughter. Through this view, our more engaging stimuli are also more comedic, which may evoke laughter and associated increases in heart rate and blood pressure (Sugawara et al., 2010). Laughter would occur at similar periods, resulting in higher cardiac synchrony. Although we do not measure respiration in the current study, previous authors find that cardiac synchrony in narrative content is not moderated by respiration (Pérez et al., 2021). We also note that laughter is observed much more frequently in groups than individually (Scott et al., 2014), and in the current study participants viewed alone. Anecdotally, we also did not detect any audible laughter during data collection and although some of our content was humorous, it wasn’t of the type that would typically induce widespread laughter.

In our study, skin conductance was not sensitive to changes in immersion. This is in contrast to other findings such as Sukalla et al. (2015) that skin conductance level relates to attentional focus and emotional engagement, making our absence of an effect intriguing. As skin conductance is directly innervated by the sympathetic nervous system, this could suggest that immersion is more tied to parasympathetic activity. Emotions linked to immersion, including awe, are theorised to be related more to parasympathetic activity than other emotions such as excitement (Shiota et al., 2011). Alternatively, it may be that our stimuli did not contain enough differences in aversive properties to evoke meaningful skin conductance differences, and that if different content were used (e.g. strong horror content), this would change. However, some findings suggest that skin conductance synchrony can be reliably induced using calming stimuli (Han et al., 2021).

## General discussion

When immersed in narratives, we allocate attentional resources to construct detailed representations of events, characters, and their intentions and emotions; to understand the content and anticipate outcomes (Zwaan, 1999). Alongside directly probing this behaviour using self-report, we may be able to index the same processes using a combination of behavioural and physiological measures. Here, we validate dual-task reaction times, heart rate, and skin conductance as measures of immersion, against self-reported engagement for a set of short video clips. In Experiment 1, we find evidence that people respond slower to a secondary task, when watching content which is rated as more engaging. In Experiment 2, we find evidence that higher self-reported engagement is related to a greater inter-subject correlation in viewers’ heart rates. We have demonstrated that it is possible to measure immersion indirectly and dynamically.

In both experiments, our measures were related to narrative engagement through the attentional and emotional dimensions of the scale. This complements existing research that film stimuli narrow attention (Bezdek & Gerrig, 2017; Bezdek et al., 2015): here, we suggest that more engaging narratives may lead to a greater narrowing of attention (indexed through slower reaction times and more synchronous heart rate).

Film has previously been considered an emotional machine (Tan, 1996) and there are accounts that fiction functions as a simulation of social worlds (Oatley, 2016). Within this framework, when engaged, viewers allocate resources towards representing and predicting beliefs, feelings, and intentions of characters. Indeed, other researchers have found that reading fiction can improve empathy and theory of mind capabilities (Black & Barnes, 2015; Kidd & Castano, 2013) and that this effect is contingent on the degree of transportation experienced (Johnson, 2012). Autonomic nervous functions, including heart rate, respiration, skin temperature, and skin conductance are similarly activated during real and imagined events (Deschaumes-Molinaro et al., 1992), further indicating that physiological responses should be sensitive to the mental simulation involved during immersion.

However, we did not find relationships between the narrative understanding or presence dimensions of the Narrative Engagement Scale. The former is consistent with previous research indicating an absence of relationship between comprehension and gaze synchrony. In a study by Hutson et al. (2017), comprehension was independently manipulated by providing some participants with narrative context and others without, but across these two conditions gaze behaviour remained similar. However, it is important to note that in our study, perceived (i.e. self-reported) comprehension is not an objective measure of understanding. Future work may choose to investigate this area further using objective metrics of understanding, for example memory, or asking participants to predict what will happen next in a scene.

The absence of a relationship between immersion and narrative presence is perhaps more surprising. Presence is generally conceptualised as an individual’s perception being oriented from the real world towards the world of the media (Waterworth et al., 2015), often described as a sense of ‘being there’ (Biocca et al., 2003), most commonly in the form of feeling spatially located within the environment (Witmer & Singer, 1998). Despite presence and immersion being used interchangeably by some authors (Nilsson et al., 2016), we did not find a relationship between our measures of immersion and self-reported narrative presence. It is possible that the feeling of being spatially located within the story world is inadequate to trigger the cognitive and emotional processes we are indexing. This could indicate a distinction between immersion as attending to and mentally representing the stimulus, and presence as feeling spatially located within the stimulus (Jennett et al., 2008). Alternatively, we may simply not have enough variability in presence ratings: note the standard deviations in narrative presence for each experiment appear meaningfully lower than other dimensions of engagement. Some researchers have argued presence is experienced as a binary rather than a continuous phenomenon (Riva et al., 2004).

In these experiments, we employed a design which was indifferent to each participant’s ratings of immersion in each clip, and individual’s propensity to become immersed. Several recent attempts have been made to classify individual’s tendency to become immersed: for example, the narrative engageability scale (Bilandzic et al., 2019), transportability scale (Dal Cin et al., 2004), and research on the link between personality traits and immersive tendency (Weibel et al., 2010). We note that future endeavours may wish to assess whether our continuous measures of immersion are contingent on participant’s individual differences, for example mental imagery abilities (Jacobs & Willems, 2018). Continuous measures may also elucidate other interesting individual factors, such as whether differences exist in the time it takes to become immersed.

As immersion itself is in part characterised by a lack of meta-awareness (Agrawal et al., 2020), reporting on the experience retrospectively within a questionnaire may be difficult (e.g. see research on the difficulty of reporting mind-wandering; Schooler et al., 2011). Despite this, we have shown several instances where behavioural and physiological measures appear to index self-reported immersion, suggesting that both measures are tapping into the underlying concept of immersion. Further, we have shown a relationship between the Narrative Engagement scale and a single-item question of immersion, suggesting internal consistency. However, some disparities between continuous and self-reported immersion exist. While our continuous measures (reaction times and heart rate synchrony) were most strongly associated with emotional engagement, single-item immersion is correlated more with attentional focus and narrative presence. As such, researchers interested in self-reported immersion may wish to incorporate additional items assessing viewers’ emotional engagement, rather than switching to a single-item question. Additionally, researchers should be aware that as questionnaires are retrospective reports, they rely on the memory of the experience. It is possible then that questionnaires are affected by primacy and recency biases (Glanzer & Cunitz, 1966), meaning that variability in immersion during the middle sections of the content is not captured.

These experiments demonstrate that reliable changes across audiences’ attention and physiology can be seen during the everyday context of watching television and film. These mediums have evolved to hold attention across extended periods, through the use of cinematic conventions and editing techniques (Cutting et al., 2010; Smith, 2012). We add to this evidence that these media are indeed well-designed to captivate viewers. That both of our experiments showed a robust relationship between continuous measures of immersion and emotional engagement suggests it is perhaps these emotional processes which predominantly immerse viewers. Future work may wish to apply our continuous measures of immersion to assess variability within a clip. For example, researchers could explore how immersion changes in response to audio-visual, editing, and narrative features. Additional file 1: Figure S5 provides an example of how ISC_HR_ may be applied to index narrative moments.

In a traditional psychology experiment, participants are often bored and disengaged and are only studied across discrete single-trial intervals. In contrast, the real world is continuous and rich in engaging stimuli. The above experiments present an opportunity to study how attention is sustained and disrupted in a naturalistic context.

## Conclusions

The purpose of the current study was to validate behavioural and physiological measures of audience immersion across several types of film and television content. Further, these experiments demonstrate the narrowing of attention and robust emotional engagement which occurs as we watch engaging media. This toolkit can now be applied to a wider range of contexts, including different media formats, or real-world situations. Our measures of immersion should additionally be sensitive to fluctuations in immersion within a single stimulus, such as a feature-length film. The dual-task paradigm and heart rate ISCs both offer the opportunity for continuous measures of audience immersion, while heart rate ISC offers this in a truly non-invasive way.

## Supplementary Information

**Additional file1**. Supplementary materials.

## Data Availability

The data sets generated and the code used to produce all figures are available in the Open Science Framework repository at https://osf.io/49xpq/.

## Footnotes

1 Note that here, and in subsequent linear mixed-effects models, a random slope was first selected over a random intercept, which yielded a singular fit. In these cases, this describes a near-perfect correlation between random intercept and slope, indicating the slope is not necessary and thus the model should be simplified.
