# Correction: Audience immersion: validating attentional and physiological measures against self-report

**DOI:** 10.1186/s41235-024-00600-7

**Published:** 2024-10-19

**Authors:** Hugo Hammond, Michael Armstrong, Graham A. Thomas, Iain D. Gilchrist

**Affiliations:** 1 School of Psychological Science, University of Bristol, 12a Priory Road, Bristol, BS8 1TU UK; 2 British Broadcasting Corporation (BBC) Research and Development, Saltford, UK

**Correction: Cognitive Research: Principles and Implications (2023) 8:22** 10.1186/s41235-023-00475-0

The original article mistakenly omitted the EPSRC funder grant number (EP/M000885/1) which the authors wish to acknowledge via this Correction article.

