# Supplementary material for: Audience immersion: validating attentional and physiological measures against self-report
Source: Cogn Res Princ Implic. 2023 Apr 19;8:22. doi: 10.1186/s41235-023-00475-0 (PMC10113978; doi:10.1186/s41235-023-00475-0)
Supplement: Supplementary file 1 — Additional file1. Supplementary materials. [file 41235_2023_475_MOESM1_ESM.docx]

**Supplementary materials**

*Supplementary Figure 1.* Counterbalancing matrix for each experiment.


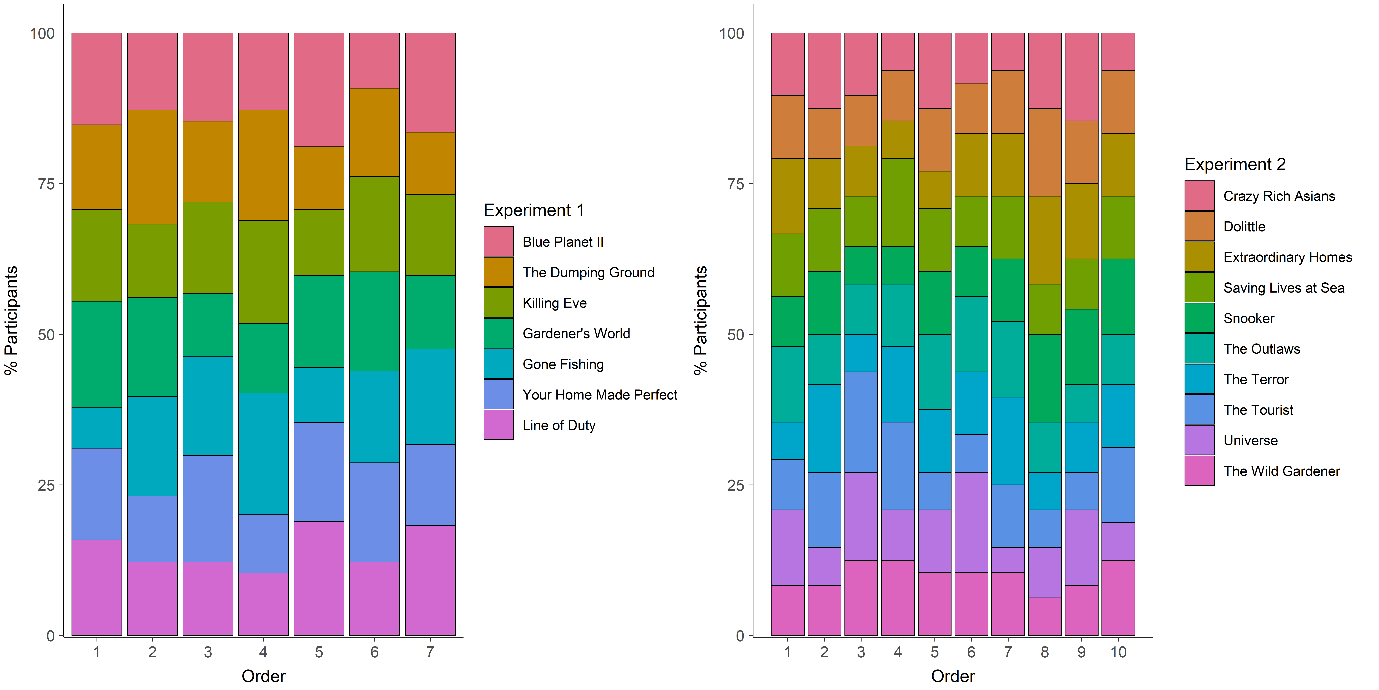


Note: This figure shows the percentage of participants who viewed each clip in each order for experiment 1 (left) and experiment 2 (right).


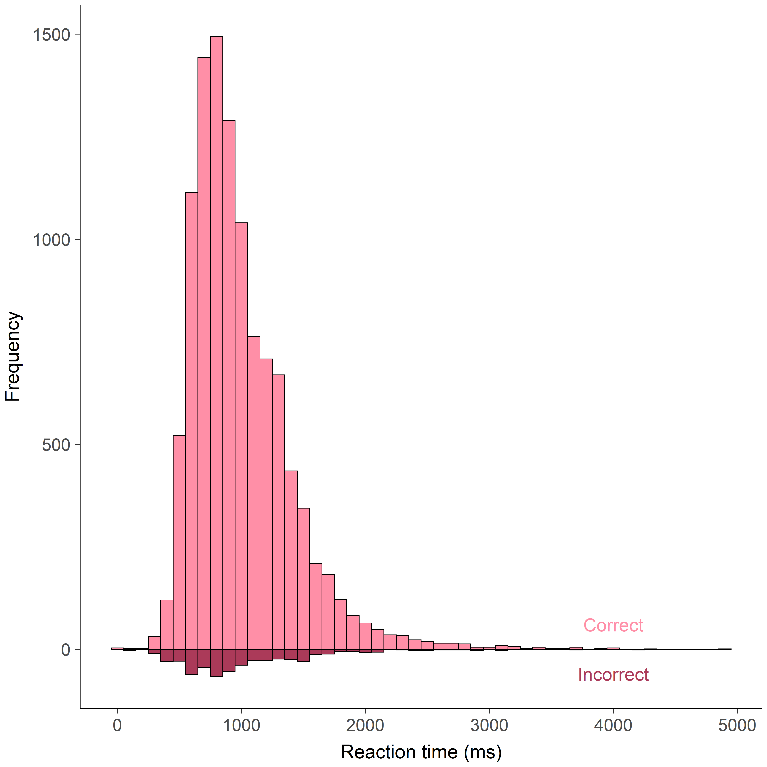
*Supplementary Figure 2.* Distribution of reaction times in experiment 1.


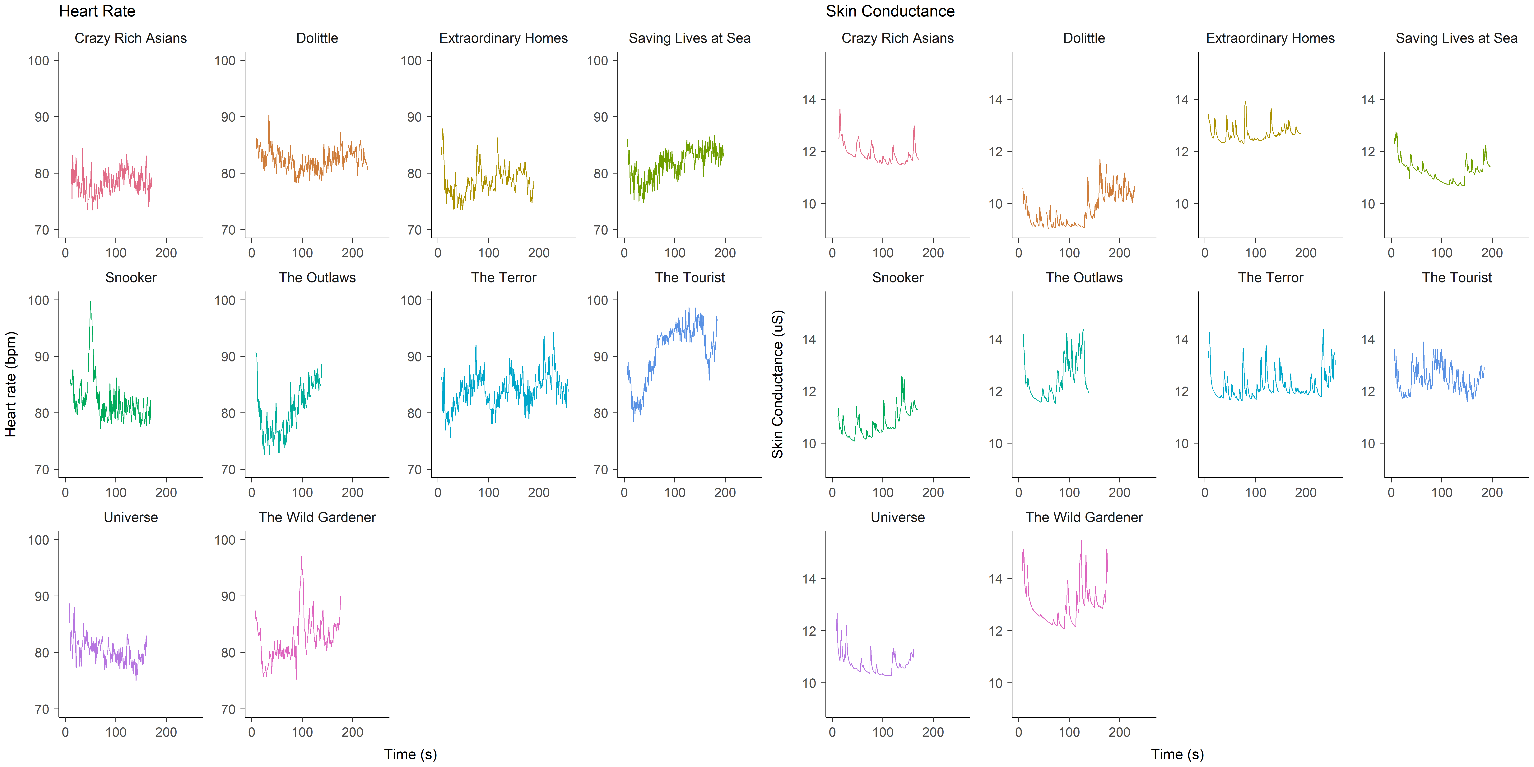
*Supplementary Figure 3.* Example time course plots of heart rate (left) and skin conductance (right) for a participant.


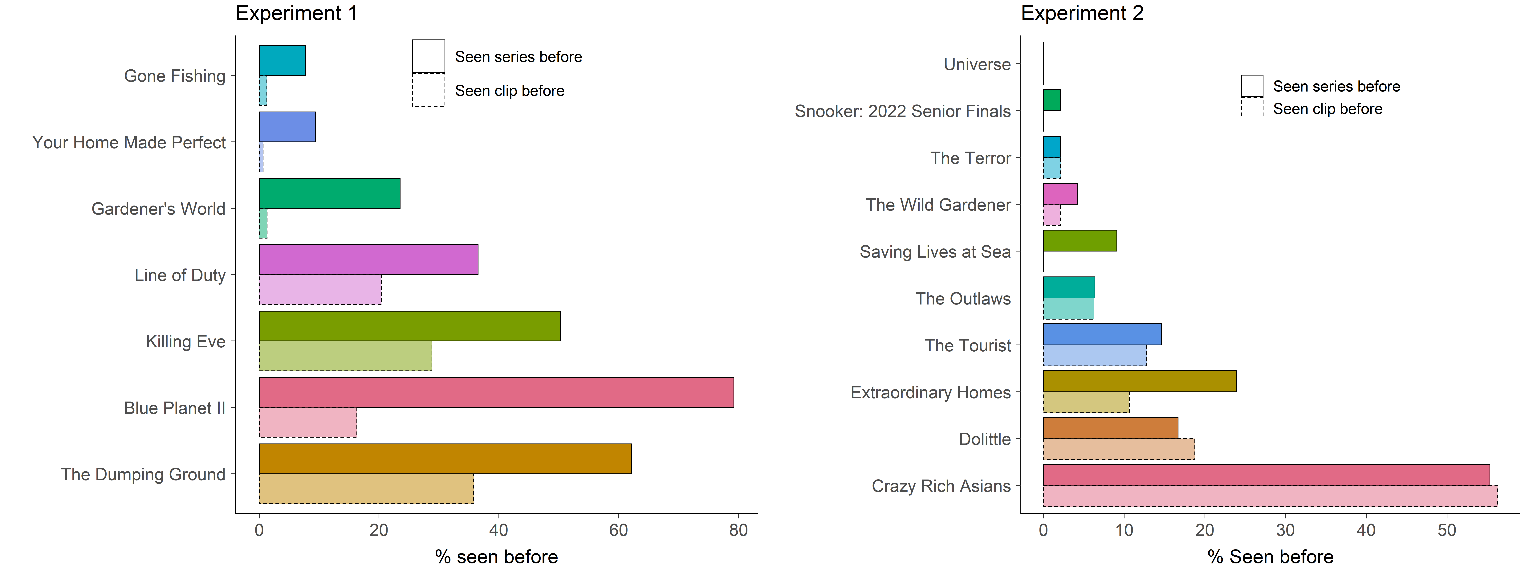
*Supplementary Figure 4.* Familiarity plots for Experiment 1 (left) and Experiment 2 (right).

Note: Each experiment asked two questions related to familiarity: ‘Have you seen **any** of the following series/film before?’ (bold outline), and ‘Have you seen the **specific clips** used in this experiment before?’ (dashed outline).

*Supplementary Figure 5.* Example of ISC_HR_ computed across time for ‘The Outlaws’.


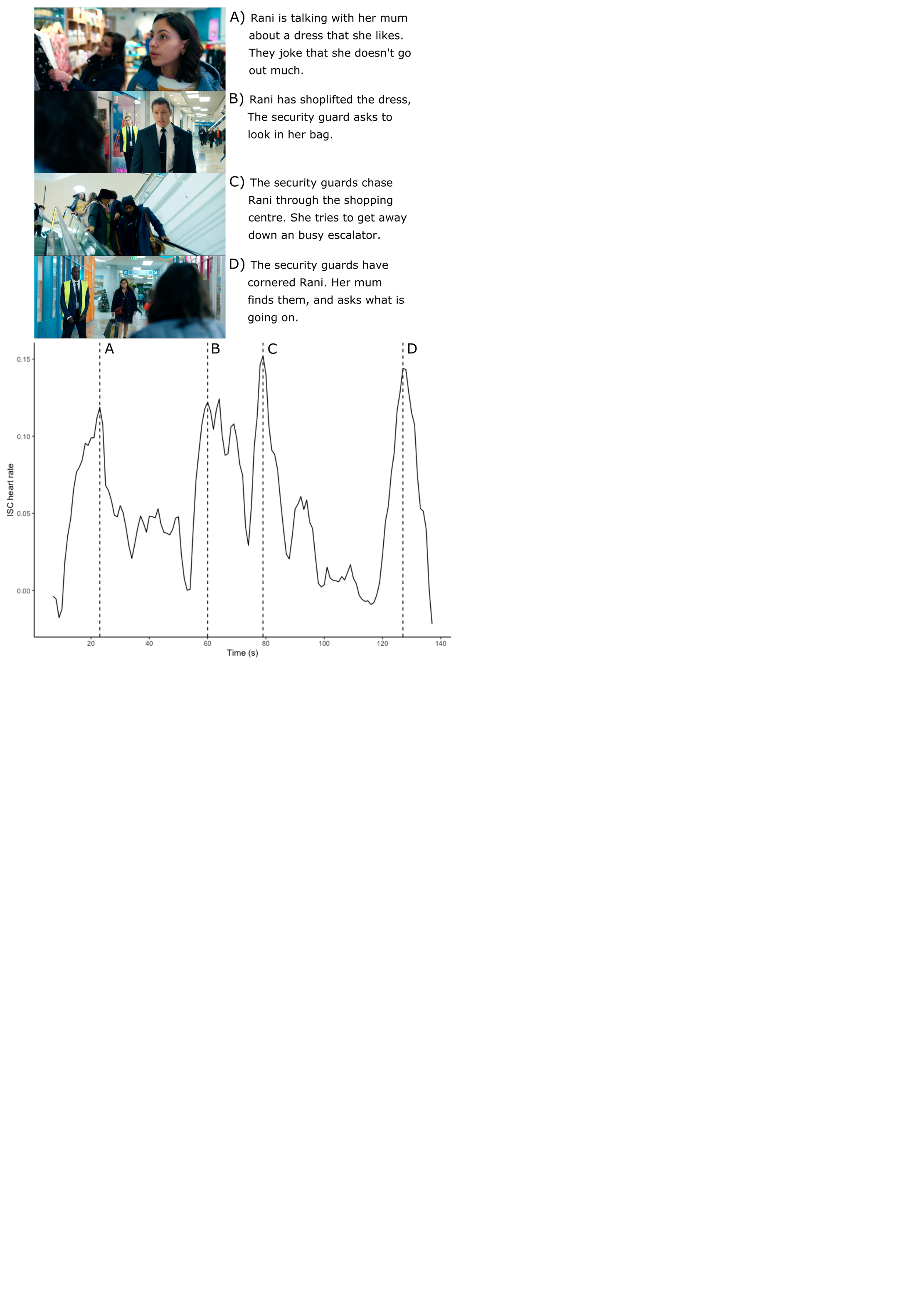
Note: For this clip, ISC_HR_ was computed using a rolling window with a width of 15 s, moved in 1 s increments rather than across the whole clip. This provides a continuous estimate of ISC_HR_ which could be linked to notable moments in the content. Here, we have identified the ISC_HR_ peaks (dashed line) and described what is occurring during those moments (A-D).

*Supplementary Materials S6.* Replication of results following pre-registered exclusion criteria.

In experiment 1, we chose to exclude participants with an average of below 50% correct responses. This deviates from our preregistered exclusion criteria of < 75% correct responses. Upon reflection, we do not think it is appropriate to exclude participants for answering incorrectly, as higher error rate may simply be a consequence of higher engagement. However, if the preregistered exclusion criteria was followed, the final sample size would be *n* = 156. Below, we present the same analyses as in the main results using the pre-registered exclusion criteria to demonstrate that the results are consistent.


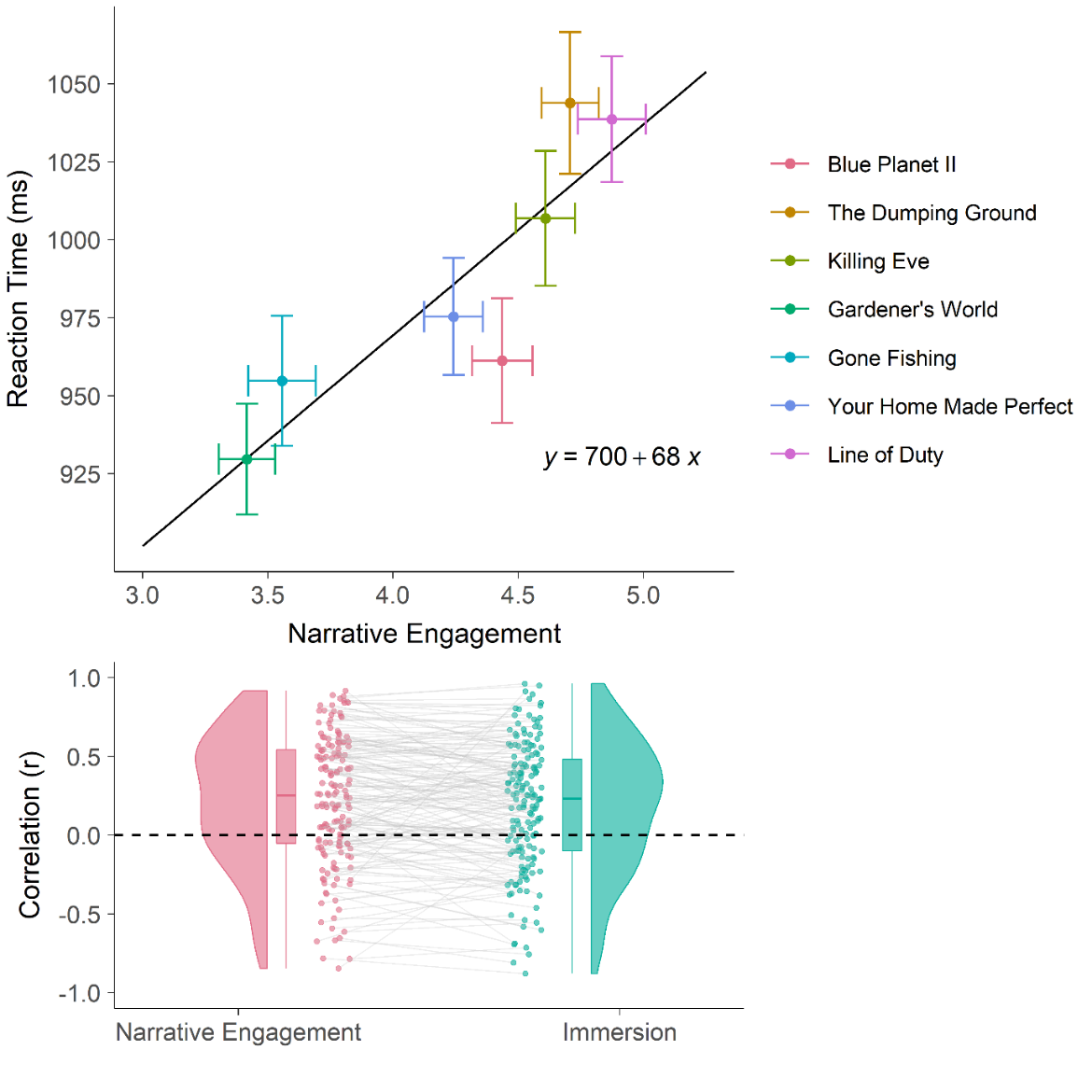


We found a significant relationship between reaction time and narrative engagement: *r*(5) = .880, *p* = .009, as well as reaction time and our single-item question of immersion, *r*(5) = .845, *p* = .017. Individual participant correlations were also significantly greater than zero for both narrative engagement (*r* = .225, *t*(156) = 7.01, *p* = 6.7 x 10^-11^) and immersion (*r* = .197, *t*(156) = 6.17, *p* = 5.64 x 10^-9^).

Exploring this relationship using a linear mixed model indicates the same trend for only emotional engagement (*b* = 15.53, 95% CI[7.30, 23.76]) and clip order (*b* = 10.95, 95% CI[6.54, 15.36]) to have a significant impact on reaction time.


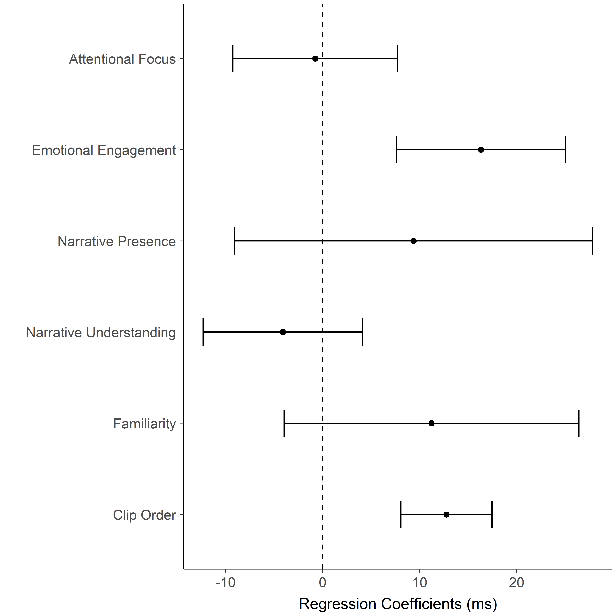


Similarly to our full analysis, ISC_RT_ was also not related to narrative engagement (*r*(5) = .438, *p* = .326), or immersion (*r*(5) = .372, *p* = .412). However, individual participant correlations between ISC_RT_ and both narrative engagement (mean *r* = .223, *t*(155) = 6.92, *p* = 1.15 x 10^-10^) and immersion (mean *r* = .194, *t*(155) = 6.07, *p* = 9.59 x 10^-9^) were significant.

*Supplementary Materials S7*. Heart rate variability analyses.

Due to dual-innervation of heart rate by sympathetic and parasympathetic activity, heart rate does not always vary predictably in response to stimuli, and as such heart rate variability is sometimes used as a measure of cognitive processing (Potter & Bolls, 2012). Using power spectral density analysis to calculate power over low and high frequency bands may address some of the issues interpreting heart rate signal. The high frequency component reflects vagal activity, whereas low frequency is argued by some to reflect exclusively sympathetic activity, and others to be composed of sympathetic and vagal tone. Consequently, researchers can calculate a ratio of low frequency to high frequency power (LF/HF) which some interpret as a measure sympatho-vagal balance (Schaffer & Ginsberg, 2017).

Here, we calculate heart rate variability for each clip using Welch’s (1967) periodogram power spectral density analysis to obtain power in the low (.04 - .15 Hz) and high (.15 - .4 Hz). Supplementary Figure 7 (left) plots the total power in each frequency domain for each clip. Supplementary Figure 7 (right) demonstrates a significant relationship between narrative engagement and LF/HF ratio (*r*(8) = .143, *p* = .003). Interpreting this as above would suggest that increases to narrative engagement results in relative lower sympathetic and higher parasympathetic activity.

However, Billman (2013) has noted several issues with interpreting LF/HF ratios: including the non-linearity of low and high frequency interactions, and the assumption that any increase to sympathetic activity will be accompanied by a reduction in parasympathetic activity. As such, we leave these analyses in the supplementary materials for readers to interpret if they wish, although advise them to do so with caution.


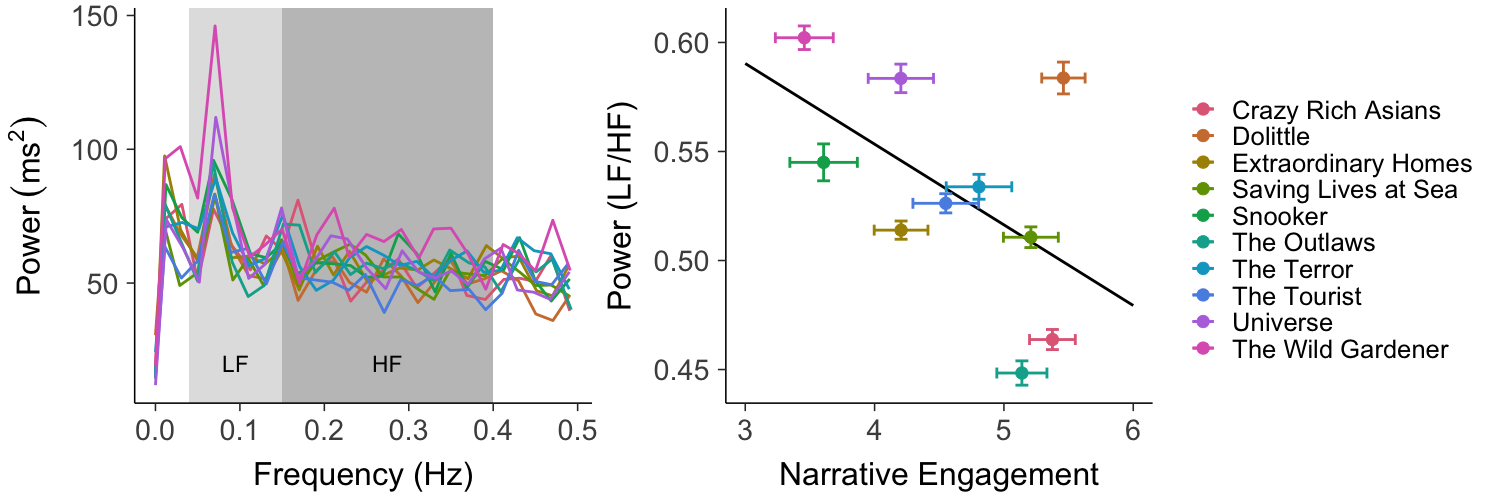


Supplementary Figure 7. Left: Total power (milliseconds squared) in each frequency domain. Right: LF/HF ratio as a function of narrative engagement.

**References**

Billman, G. E. (2013). The LF/HF ratio does not accurately measure cardiac sympatho-vagal balance. *Frontiers in physiology, 4*, 26.

Potter, R. F., & Bolls, P. (2012). *Psychophysiological measurement and meaning: Cognitive and emotional processing of media*. Routledge.

Shaffer, F., & Ginsberg, J. P. (2017). An overview of heart rate variability metrics and norms*. Frontiers in public health*, *258*.

Welch, P. (1967). The use of fast Fourier transform for the estimation of power spectra: a method based on time averaging over short, modified periodograms. *IEEE Transactions on audio and electroacoustics, 15*(2), 70-73.
